# Investigating the factors of enterprise social media strain: The role of enterprise social media’s visibility as a moderator

**DOI:** 10.1371/journal.pone.0264726

**Published:** 2022-03-08

**Authors:** Ying Li, Abdul Hameed Pitafi, Haoning Li

**Affiliations:** 1 School of Economics, Tianjin University of Commerce, Tianjin, China; 2 Department of Computer Science, Sir Syed University of Engineering and Technology, Karachi, Pakistan; University of Education, PAKISTAN

## Abstract

The significant effect of enterprise social media (ESM) usage has been extensively researched. However, recent studies and analysis have also emphasized the importance of understanding the negative aspects of ESM’s use. By applying uses and gratifications theory (UGT), this study proposes a research model that tests how employees’ ESM usage (hedonic, social, and information values) leads to ESM-related strain through perceived information overload. The study collected data from 315 Chinese employees using a survey method and analyzed the results using AMOS 21.0 software. Structural equation modeling (SEM) was applied to analyze the proposed hypothesis. The results indicate that perceived hedonic, social, and information values are significant predictors of perceived information overload. Such overload is also significantly associated with ESM-related strain. The results also indicate that ESM visibility strengthens the significant relationship between perceived information overload and ESM-related strain. Furthermore, managers can also train individuals to use ESM appropriately. We recommend that employees can better control and manage their ESM usage by recognizing the causes of excessive use.

## 1. Introduction

Enterprise social media (ESM) has introduced modern management practices to organizations, from the development of creative marketing plans to the transformation of connectivity, cooperation, and information exchange. An extensive literature shows that applying ESM to the workplace can improve individual job efficiency and productivity [1–5]. According to Leonardi and Meyer [4], ESM is a digital portal that allows individuals to share expertise with specific workers, to broadcast detailed information to everyone, and to edit, filter, and access the content of others without the interdependencies of time and space. Previous research has found that proper use of ESM by individuals can benefit both workers and organizations [6, 7]. However, it can be detrimental if its use is excessive. Therefore, as ESM becomes more widely used in the workplace, workers may be overwhelmed with information, interaction, and social messaging, resulting in perceived overload and strain. For example, excessive use of ESM can lead to information overload and irritation, to individuals not concentrating on their task, and to errors in decisions. In particular, inappropriate and unreasonable use of ESM is becoming common among employees, with significant consequences for individuals and organizations. Therefore, to avoid and reduce the harmful consequences of inappropriate ESM use, it is critical to recognize the predictors and root causes of such activities—the motivation for our research.

According to recent literature, individuals may utilize specific technologies or social media to satisfy their desires or needs; if these incentives or necessities are gratified, they will prefer to re-experience them [8, 9]. ESM, as a social platform, encourages employees to participate in a variety of tasks such as posting, gathering information, and interacting with others [10, 11]. These practices satisfy individuals’ unique needs—interpersonal, informational, and hedonic—through ESM [12, 13]. Alksasbeh, Abuhelaleh [14] found that, when social media addresses the expectations of students, it can increase their level of its use. Furthermore, several researchers have discovered that the fulfillment of needs can influence users’ inappropriate usage [15, 16]. Thus, as individuals feel that their requirements are addressed by social media, they increase their use of the technology to gain additional satisfaction. When the intensity of use reaches a certain level, information overload occurs [12, 17]. “Information overload” is when the amount of information to which individuals are subjected exceeds the degree to which they can manage it efficiently [18]. As a result, the present study investigates the relationship between the gratification of needs—the needs satisfied by ESM usage—and perceived information overload.

However, the impact of perceived information overload on ESM-related strain is not independent of the ESM context. In comparison to other communication technologies, ESM provides a forum for open employee communication and cooperation [19]. It makes conversations between workers visible to all in the organization [20]. On the one hand, ESM visibility offers extremely visible communication among workers that can promote social bonding, social relationships, and information sharing [3]. On the other hand, ESM visibility offers uncontrolled and unorganized content that can overwhelm individuals’ interpretive and analytical abilities, [21] resulting in information ambiguity. Therefore, highly visible information on the ESM platform may also interrupt employees’ daily life and cause strain. For example, when individuals search for information using ESM to address problems, they may deal with a huge quantity of information [22] which contributes to ESM-related strain. However, although recent studies have clearly considered ESM visibility for its logical context, it has not been empirically examined much [21]. To address this research gap, this research explores the moderating role of ESM visibility on the relationship between perceived information overload, and ESM-related strain.

**Fig 1:**
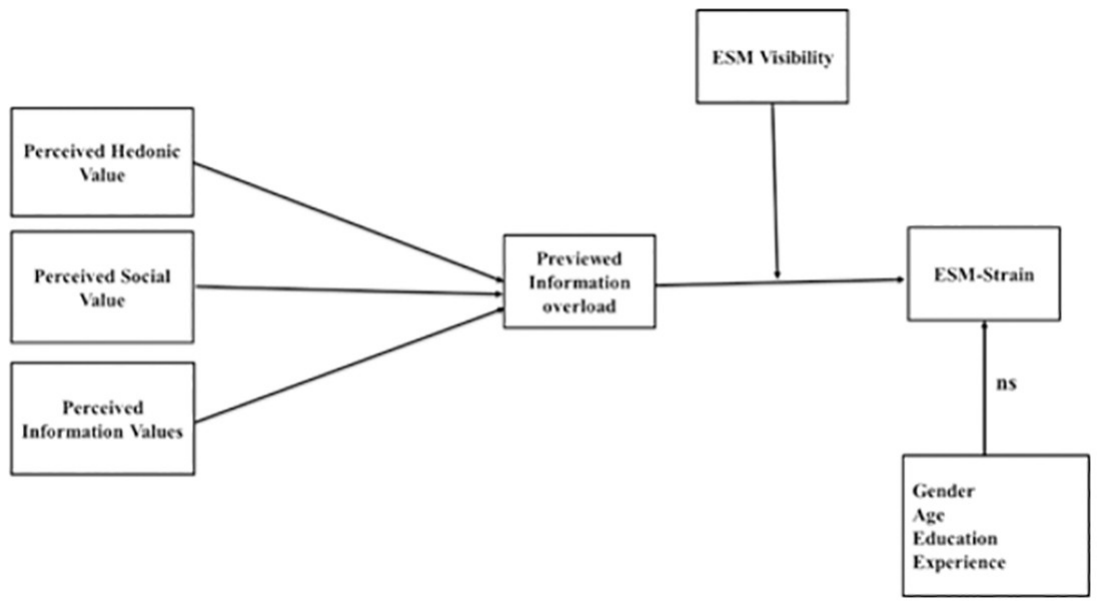
Conceptual model.

The purpose of this study is to examine the relationship between the gratification of needs and ESM-related strain through perceived information overload using data collected from Chinese employees. Based on uses and gratifications theory, this study also investigates the moderating role of ESM visibility in the relationship between perceived information overload and ESM-related strain. This study makes an important contribution to the current literature. It firstly highlights the relationship between the gratification of needs and perceived information overload to address the negative impact of social media usage. Secondly, it discusses the potential role of ESM visibility as a moderator. Thirdly, the results of this research may help managers and ESM designers to better understand the causes of ESM-related strain and perceived information overload. This study extends our understanding of the relationship between ESM usage (for hedonic, social, and information value) on strain and offers evidence that allows managers to design ESM usage guidelines for employees to control the negative consequences of unreasonable ESM usage. Fig 1 indicates the conceptual model of the study.

## 2. Theoretical background and literature review

### 2.1 Uses and gratifications theory (UGT)

Uses and gratifications theory (UGT) provides a useful framework for understanding why individuals use and choose a certain type of technology [23, 24]. The basic assumption of UGT is that people are not automatically attracted to media content but instead utilize media to satisfy their different hedonic, emotional, and psychological needs [25]. UGT can also be used to describe user attitudes in computer-mediated communication (CMC) media contexts [26]. Unlike conventional media, such as print and television, CMC enables users to personalize their information to communicate with others. In the context of UGT, scholars argue that individuals initially use media or technology to fulfill their requirements and, after satisfaction, they continue to repeat the same experience [23, 25]. UGT could thus provide a suitable theoretical framework for our study.

UGT has been commonly used to describe CMC media use in a wide range of contexts, such as online gaming [27], internet services [28], and email [29]. In recent technology, scholars have also applied UGT to social media technology such as Facebook, WeChat, Twitter, and Weibo [24, 30]. For example, Gan and Li [30] investigated three types of gratification that would encourage people to continue using WeChat: hedonic, social, and utilitarian. Based on the UGT and ESM usage literature, we propose that ESM would provide workers with three types of gratification: social, information, and hedonic [8, 23]. *Social* gratification refers to the use of ESM by individuals to establish social relationships with coworkers, allowing them to recognize the social value provided by ESM [23, 30]. *Information* gratification is the fulfillment of information requirements. Employees may access a range of information from ESM technology—such as content, and information references—allowing them to understand the importance of the information provided by ESM, thereby gratifying their need for information [23, 30]. *Hedonic* gratification allows employees to use ESM to obtain enjoyment and pleasure, thereby accessing its hedonic value [23, 30]. Although several scholars have recognized different forms of gratification offered by ESM for individuals, and others have stressed the links between social media availability and the gratification of needs, very little research has been conducted on the connection between perceived information overload and ESM-related strain. To fill this research gap, we used UGT as the theoretical foundation to investigate the workplace link between the gratification of needs and ESM strain through perceived information overload.

### 2.2 ESM visibility

ESM visibility, which identifies the specific visibility features offered by digital media technology such as ESM, is essential to ESM technology [31]. This visibility reflects the accessibility of workplace communication between individuals for a wider organizational audience [19, 32]. Before the rapid development of ESM technology, workplace conversations among colleagues which are related to the work environment were long kept private [18, 33]. ESM differs from previous communication technologies because it enables an individual to view communication between colleagues, even if that individual is not personally engaged in that communication [3, 34, 35]. Accordingly, the communication of content between two colleagues on an ESM platform can often occur on the wall of an employee who is not overtly involved in that communication session [36, 37]. According to Treem and Leonardi [32], ESM visibility enables unparalleled knowledge by employees of their coworkers’ actions by allowing them to view the content of their colleagues’ communication.

Several studies have shown that ESM visibility improves employee information exchange and social engagement within the organization by increasing the accuracy of workers’ meta-knowledge [38, 39]. Scholars have also identified negative consequence of ESM visibility, which include interruptions, violation of privacy, and information overload [17, 40]. Theoretical literature indicates that ESM visibility may play a dual role in the workplace. On the one hand, ESM visibility presents workers with the rapid ability to identify the expertise of coworkers in a specific domain and discover mutual preferences and hobbies [41, 42]. On the other hand, ESM visibility encourages workers to view excessive amounts of information beyond their capacity to process [43]. As a result, this study examines the impact of ESM visibility on the relationship between perceived information overload and ESM strain.

### 2.3 ESM-related strain

Several studies have recently investigated ESM usage in the workplace with mixed findings [12, 17, 44, 45]. For example, Chen and Wei [17] reported that social or work-related use of ESM causes overload and ESM-related strain among employees; this may have an adverse impact on their work efficiency. Cao and Yu [12] examined the link between excessive ESM use and work outcomes, observing that such excessive use has adverse effects on job performance. In contrast, Pitafi, Kanwal [44] reported that ESM use has a significant impact on employee work performance. Hence, theoretical studies have begun considering the negative aspects of ESM technology, such as ESM-related strain. Since each user communicates to other users throughout the ESM network, a flood of communication or information is produced. This high-speed flow of information may create ESM-related strain among employees that can include feelings of anxiety, pressure, helplessness, and tension. Ayyagari, Grover [46] reported that excessive use of technology may influence employees’ work outcomes and, ultimately, contribute to strain. As a result, this study examines the ESM usage factors that cause ESM-related strain among individuals.

## 3. Hypothesis development

### 3.1 Users and gratifications theory and perceived information overload

“Hedonic ESM usage” applies to the use of ESM technology primarily for entrainment and pleasure [12]. “Perceived hedonic value” signifies the satisfaction and happiness that results from the ESM content and the connections thereby established with other individuals [24]. To realize hedonic value, employees may experience satisfaction, enthusiasm, tweets, and excitement with coworkers [23, 47]. The intrinsically enjoyable existence of ESM encourages greater employee engagement and its extensive use [48, 49]. Consequently, an ESM participant may be excited because they discover similar preferences or read interesting material shared by colleagues [8, 50]; they may then share more content. In order to sustain a high level of pleasant experience, these workers can behave irrationally, expending much time and energy on ESM and exchanging ever more information [12, 51]. Since each individual is sharing interesting items with other individuals on the ESM platform, there is a stream of information. This rapid flow of information can result in information overload. As a result, based on the literature, we suggest the following hypothesis:

**H1a: The use of ESM for perceived hedonic value has a positive effect on perceived information overload.**

“Perceived social value” refers to the benefits that one may experience by efficiently establishing or managing personal relationships, seeking social support, and promoting social interaction with coworkers on the ESM platform [12]. ESM is a web-based platform that links people with family members, relatives, acquaintances, and coworkers at any time and from any location [4]. As the number of social connections increase, workers may receive a significant number of responses from their online friends through ESM [9]. In order to sustain these huge social networks for acquiring social support and a sense of belonging, individuals must respond as quickly as possible by ESM [12]; this type of action can result in information overload. According to UGT, as workers consider the social value provided by using ESM at workplace, their usage can be affected. In other words, they can increase their use of this technology as a result of the socially significant benefits they receive. Information overload can occur when this rate of use reaches its maximum. Sun, Wang [52] discovered that social importance greatly increases the degree to which people use ESM. Chen and Kim [15] have shown that the social importance perceived by employees is a significant factor in supporting ESM use. We thus propose:

**H1b: The use of ESM for perceived social value has a positive effect on perceived information overload.**

ESM serves as a valuable channel for communicating and exchanging information [53, 54]. “Perceived information value” refers to the benefits of a user obtaining important information from ESM [55]. Previous studies have reported a significant correlation between perceived information value and the actual use of technology [56]. ESM encourages individuals to post and share work-related information, allowing employers to satisfy their workers’ information needs [31, 57]. Additionally, employees’ information value expectations can affect information exchange [24]. For example, an employee with a high perception of information value is more inclined to share information, ideas, and interactions on the ESM platform with colleagues. Consequently, they may expend much time and energy checking for notifications and sharing material on ESM [35, 44]. The more valuable the information an employee obtains from the online community, the more likely they will share information in that community. According to recent research, information overload is exacerbated by individuals’ communication, content-sharing, likes, updates, comments, and posts on an ESM platform [58]. Information value also motivates employees to share more information. Therefore, this study proposes the following hypothesis:

**H1c: The use of ESM for perceived information value has a positive effect on perceived information overload.**

### 3.2 Perceived information overload and ESM-related strain

Much data has been generated with recent advances in information and communications technology; consequently, the phenomenon of information overload has become more readily recognized and encountered [12, 17]. “Information overload” refers to people’s assessment and interpretation of the types of items that are outside their ability to manage [59]. Scholars have used overload terms in several fields of research, including “work overload” [46], “information overload” [59], and” system-feature overload”. As volumes of information cross a certain threshold, people may have problems locating and interpreting it [36] and thus make decision errors. Therefore, when an employee seeks additional information that is needed, their decision-making ability may suffer.

The proliferation of ESM technology has resulted in massive amounts of information being immediately generated and disseminated [55]. ESM is a public platform, so employees can post, broadcast, exchange, and disseminate information rapidly at any time [19, 60]. As a result, they are unable to process information efficiently, indicating that excessive use of ESM can result in information overload [12]. According to Wurman [61], information overload causes individuals to feel depressed, uneasy, and emotionally exhausted. Zhang, Zhao [59] also found that a high volume of information may contribute to social network exhaustion as the rapid production and dissemination of information on ESM has negative effects such as fear, frustration, and anxiety. This study therefore suggests the following hypothesis:

**H2: Perceived information overload has a positive effect on ESM-related strain.**

### 3.3 ESM visibility as moderator

ESM visibility allows individuals to make their behaviors, information, and knowledge visible to their colleagues [31, 62, 63]. ESM visibility presents a quick way of identifying what other employees are doing and with whom they are communicating [3, 35, 36]. ESM visibility allows them to easily approach the broad social network. Such unregulated interactions may increase the possibility of information overload. Earlier studies have concluded that ESM visibility may contribute to information overload because there is a vast amount of information outside of an individual’s control [12, 22]. For example, notifications and a constant flow of work- or non-work-related information require employees to view and process a considerable amount of information with workmates. This unstructured flow of information can divert their focus from their work and lead to negative attitudes toward others.

Furthermore, the visibility of ESM leads employees to be anxious about the disclosure of their weakness and failures; they may believe that ESM usage requires a significant amount of time and effort, thus raising their loading perception [17, 64]. ESM visibility thus amplifies the flow of information: it encourages employees to establish a better social presence on ESM, which necessarily requires workers to share and exchange a significant amount of information with other colleagues [65], leading to information overload. Although ESM allows workers to collaborate and fulfill their workplace and social needs [66, 67], the visibility it facilitates causes an intensifying of demands, creating a feeling of overload and fatigue [68]. Therefore, this study proposes the following hypothesis:

**H3: ESM visibility moderates the significant relationship between perceived information overload and ESM-related strain, such that the higher the ESM visibility, the higher the relationship between perceived information overload and ESM strain.**

## 4. Research methods

### 4.1 Data collection procedures

In order to achieve its aim, this study collected data by surveying Chinese workers employed in several companies. Due to the increasing popularity of ESM technology in China, we decided to conduct the survey there. Moreover, ESM technology has been extensively adopted by many businesses as a cost-effective tool for their employees’ work-related communication. To capture accurate and valid responses, the current research focused on specific information about ESM usage in the workplace, making data collection through a survey difficult. Consequently, we collaborated with a well-known educational institution to ensure the reliability of our study. This organization is involved in several training programs for employees, especially about information systems. We selected organizations that had adopted ESM technology for their employee’s work-related communication. Before the data collection, we also conducted several meetings with employees to ensure their use of ESM. Several additional strategies were used to identify valid responses; for example, we developed two questions to distinguish invalid answers. The nature of these questionnaires corresponds to the two elements in the questionnaire but contradicted the original interpretation of the items. If the participants gave the same answers to the two adjacent questions, then their replies were considered irrelevant. If the time it needed to complete the questionnaire was quite small, it was declared a non-serious response and was removed. We also discussed the objective of study with the managers of the selected companies and assured employees that their feedback would remain confidential, only to be used in academic research. Before data collection, we designed the questionnaire and invited five PhD-level faculty members of the information systems department for review and suggestions. After this discussion and feedback, some items of the questionnaire were redesigned. We also conducted a pilot study on 57 participants; its results were found accurate, such that >0.700. The results of the pilot study motivated us to collect further data. In addition, authors has followed the ethical guidelines of Tianjin University of Commerce China. This study has approved by the ethical committee of Tianjin University of Commerce China. The ethical committee also has waved the consent of this study.

Over August to November 2020, the survey questionnaire was distributed to employees. To encourage the response rate, we sent reminder emails and made phone calls to all the participants. We sent 450 questions and received 340 responses—a response rate of 75.55% within four months. After evaluating 315 complete questionnaires used in final data set, some were discarded because they were improperly completed or some entries were left blank. Furthermore, following the procedure of Armstrong and Overton [69], we used the chi-squared procedure to analyze possible nonresponse bias by comparing the first and last 25% of participants over all indicators. This found that the two groups did not vary substantially, indicating that nonresponse bias was not a major problem. The demographic details for the survey are shown in Table 1.

**Table 1:** Demographics.

Variables	N	Percentage	Variables	N	Percentage
**Gender**			**Qualification**		
Male	187	58.40	Under-graduate	95	30.20
Female	128	40.60	Graduate	100	31.70
**Age**			Masters or Above	120	38.10
Between 21–30	78	24.80	**Experience**		
Between 31–40	88	27.90	Less than- 1 year	97	28.60
Between 41–50	89	28.30	1–2 years	99	29.20
>50 year old	60	19.00	>2years	113	42.20

### 4.2 Research instruments

Previously validated instruments were used in this study to analyze the perceptions of participants. All the constructs were measured using a five-point Likert scale which ranged from “strongly agree” to “strongly disagree”. Since this research is based on Chinese workers, the we adopted recommendations from previous studies [70] and used a back-translation mechanism to ensure the accuracy of all instruments. Firstly, we invited three experienced native-speaking Chinese translators to translate the original English version of the questionnaire into Chinese. We then approached three other Chinese professionals to translate the Chinese version of the questionnaire into English; this procedure was replicated several time times before the translation accurately reflected the original items. A total of ten constructs were used in this study, including the control variable. The details of all the measurement items follow.

**ESM-strain.** The outcome construct of ESM-strain included four items and was measured using items from Ayyagari, Grover [46]. The scale measures the overall strain with excessive use of ESM. The sample item of this scale is “I feel drained by activities that require me to use enterprise social media”.
**Perceived information overload.** The scale of this overload consisted of four items and was devised using items from Zhang, Zhao [59]. This scale measured the overall information load with excessive use of ESM. The sample item of this scale is “I am often distracted by the excessive amount of information available to me on enterprise social media”.
**ESM visibility.** ESM visibility was used as a moderator construct and consisted of three items. ESM visibility was measured using items from Leonardi [19]. The sample item of this scale is “Enterprise social media enable me to see other coworkers ‘answers to other coworkers’ questions”.
**Perceived social value.** The scale of perceived social value consisted of three items and was measured using items from Ding, Yang [23] and Zhang, Li [56]. The sample item of this scale is “Sharing information with others using ESM can improve my relationship”.
**Perceived information value.** The scale of perceived information value consisted of four items and was measured using items from Ding, Yang [23] and Zhang, Li [56]. The sample item of this scale is “I accumulate much knowledge through ESM users’ shared information”.
**Perceived hedonic value.** The scale of perceived hedonic value consisted of three items and was measured using items from Ding, Yang [23] and Zhang, Li [56]. The sample item of this scale is “I have fun interacting with ESM”.
**Control variables.** In order to analyze the actual effect of an independent variable on a dependent variable, we also controlled some constructs that may affect the outcome. Following the guidelines of previous studies, gender, age, education, and experience were used as control variables [71].

## 5. Results and analysis

Before being analyzed, all data was screened using SPSS software for gaps or outliers in the data set. We analyzed the data in two steps. Firstly, we tested reliability, standard factor loading, and the validity of all the instruments. Secondly, we applied structural equation modeling to analyze the hypothesis of the study.

### 5.1 Measurement model

AMOS and SPSS assessed the reliability, convergent validity, and discriminant validity of the proposed research model. By applying a two-step approach, we initially conducted confirmatory factor analysis (CFA) to test the measurement model and determine the reliability and validity of the research model before analyzing the structural relationship of the suggested hypotheses. Previous studies recommended that the values of Cronbach’s alpha (CA) and composite reliability (CR) should be higher than the minimum suggested value of 0.700 [72–74]. The findings of Table 2 indicate that the CA values of all the constructs range from (0.770 to 0.895) and the CR values from (0.840 to 0.889)—higher than the suggested value of 0.700. The average variances extracted (AVE) of all the constructs are also shown in Table 2; they range from (0.611 to 0.715), higher than the recommended value of 0.500 [72, 75, 76]. Similarly, the literature recommends that the loading of all the items should be higher than 0.600 [72], and the results of Table 4 indicate that all the items have loadings higher than 0.600. Thus, all of the findings indicated that the research model has an appropriate degree of convergent validity and reliability.

**Table 2:** CFA analysis.

Constructs	Items	Cronbach α	Composite Reliability	AVE	MSV	ASV
Perceived Social Value	3	0.770	0.881	0.713	0.110	0.061
Perceived Information Value	4	0.866	0.862	0.611	0.135	0.051
Perceived Hedonic Value	3	0.858	0.840	0.637	0.275	0.120
ESM-Strain	4	0.859	0.862	0.611	0.135	0.046
Perceived Information overload	4	0.895	0.889	0.669	0.275	0.136
ESM visibility	3	0.853	0.882	0.715	0.197	0.102

Note: AVE = average variance extracted; MSV = maximum shared variance; AVE = average variance extracted; ASV = average shared variance; discriminant validity = ASV < MSV.

We used several methods to analyze the discriminant validity of the proposed research model by observing the results of Tables 2–4. The results of Table 2 indicate that all constructs have MSV values higher than the ASV values [36]. We then applied the procedure suggested by Fornell and Larcker [72] to analyze the discriminant validity of the research model; Podsakoff, MacKenzie [77] suggested that the highest co-relation value between variables should be less than 0.700. Next, we compared the pair-wise square root of the AVE of all the constructs with inter-correlation in Table 3. The results indicate that all the AVE square-root values are higher than the inter-correlation values, suggesting an adequate level of discriminant validity for the proposed research model. In addition, we also considered the results of Table 4, which indicated that the value of each item on its assigned construct was higher than that of the other construct. Hence, we conclude that the research model also has an acceptable level of discriminant validity.

**Table 3:** Correlation matrix and mean, standard division.

Construct	Mean	SD	1	2	3	4	5	6	7	8	9	10
1. Perceived Social Value	3.031	0.943	**0.844**									
2- Perceived Information Value	4.038	0.601	0.088	**0.781**								
3- Perceived Hedonic Value	3.553	0.791	0.331**	0.191**	0.798							
4- ESM-Strain	3.998	0.655	0.019	0.368**	0.088	**0.781**						
5- Perceived Information overload	3.643	0.770	0.321**	0.259**	0.524**	0.200**	**0.817**					
6- ESM visibility	3.720	0.831	0.286**	0.099	0.415**	0.218**	0.444**	**0.845**				
7- Experience	**NA**	**NA**	0.015	0.010	-0.100	0.026	-0.054	-0.012	**NA**			
8- Education	**NA**	**NA**	0.090	0.041	0.040	-0.106	0.108	-0.072	0.463	**NA**		
9- Age	**NA**	**NA**	-0.037	-.010	0.084	0.021	0.017	0.012	-0.083	-0.053	**NA**	
10- Gender	**NA**	**NA**	-0.044	-0.111	0.036	-0.021	0.068	0.110	-0.022	-0.016	0.024	**NA**

Note:

*p<0.05,

**p<0.01.

**Table 4:** Cross-loading.

Construct	Items	PIO	PIV	ESM-S	ESMV	PSV	PHV
Perceived Information Overload **(PIV)**	**PIO1**	**.915**	.137	-.014	-.089	-.083	-.058
	**PIO2**	**.880**	-.077	.028	-.107	-.049	.081
	**PIO3**	**.774**	-.037	-.012	.138	.097	-.069
	**PIO4**	**.683**	.020	-.015	.127	.078	.069
Perceived Information Value (**PIV)**	**PIV1**	-.101	**.828**	.027	.051	.009	.031
	**PIV2**	.070	**.818**	-.053	.102	.020	-.052
	**PIV3**	.014	**.801**	-.010	-.005	.013	.058
	**PIV4**	.073	**.670**	.084	-.163	-.038	-.007
ESM-Strain (**ESM-S)**	**ESM-S1**	-.024	.024	**.879**	.083	.000	-.091
	**ESM-S2**	-.068	.051	**.818**	-.012	-.018	.062
	**ESM-S3**	.126	-.031	**.745**	-.018	.016	.001
	**ESM-4**	-.027	-.006	**.670**	-.021	.015	.007
ESM visibility **(ESMV)**	**ESMV1**	-.006	.023	-.071	**.893**	-.125	.077
	**ESMV2**	.024	-.094	.041	**.876**	.019	-.051
	**ESMV3**	-.052	.087	.065	**.761**	.064	.004
Perceived Social Value (**PSV)**	**PSV1**	-.010	.069	-.062	-.011	**.888**	.000
	**PSV2**	.015	-.018	-.048	.093	**.822**	-.050
	**PSV3**	-.021	-.042	.117	-.126	**.821**	.073
Perceived Hedonic Value **(PHV)**	**PHV1**	-.047	.112	-.019	-.005	-.042	**.866**
	**PHV2**	-.014	-.003	-.065	-.020	.087	**.789**
	**PHV3**	.133	-.118	.089	.090	-.012	**.734**

Common method bias (CMB) may occur in the responses due to the existence of cross-sectional data [77]. For this research, we used a multipronged method to assess the probability of CMB. Firstly, we attempted to minimize the possibility of CMB at the participant level by using one reverse item to keep the participants focused when responding to the questionnaire. Secondly, to examine the probability of CMB in the data set, we used Harman’s single-factor test [78, 79]. The results indicated that there were six variables with eigenvalues greater than 1.0—the first factor only indicated 26.59% of the variance, which was less than the 40% threshold. Thirdly, the findings of Table 3 confirmed that all of the constructs have co-relation values smaller than 0.600 [80]. In addition, we used the approach developed by Liang, Saraf [81] to analyze the CMB concern. As a result, we examined the substantive factor loading and method factor loading for each variable. The findings revealed that the substantive factor accounted for 66.3% of the variance, while the method factor accounted for 1.3%, indicating that there was no likelihood of an issue with CMB in the existing study. Finally, we performed a variance inflation factor (VIF) procedure to analyze the possibility of CMB. The outcome revealed that VIF results are lower than the minimum suggested value of 3.3 [82], meaning that CMB is not a significant problem in this analysis. Altogether, the evidence demonstrated that there was no CMB problem in the current study.

Prior to assessing the structural equation modelling, the fit values of the measurement model were analyzed using AMOS version 21.0 with a maximum likelihood estimation method for all variables [83]. The outcome indicated that the values of model fit (CFI = 0.910, TLI = 0.890, IFI = 0.911, NFI = 0.874, PNFI = 0.873, REMSA = 0.053, CMIN/DF = 3.083) were within the suggested range and satisfactory, as indicated in Table 5.

**Table 5:** Comparison measure model and structural model.

	Absolute fit measures			Incremental fit measures		Parsimonious fit measures		
Model	X ^2^/DF	SRMR	RMSEA	NFI	PNFI	CFI	IFI	TLI
MM	3.083	0.053	0.078	0.874	0.873	0.910	0.911	0.890
SEM	3.520	0.061	0.079	0.870	0.870	0.903	0.903	0.885

### 5.2 Structural model

**Fig 2:**
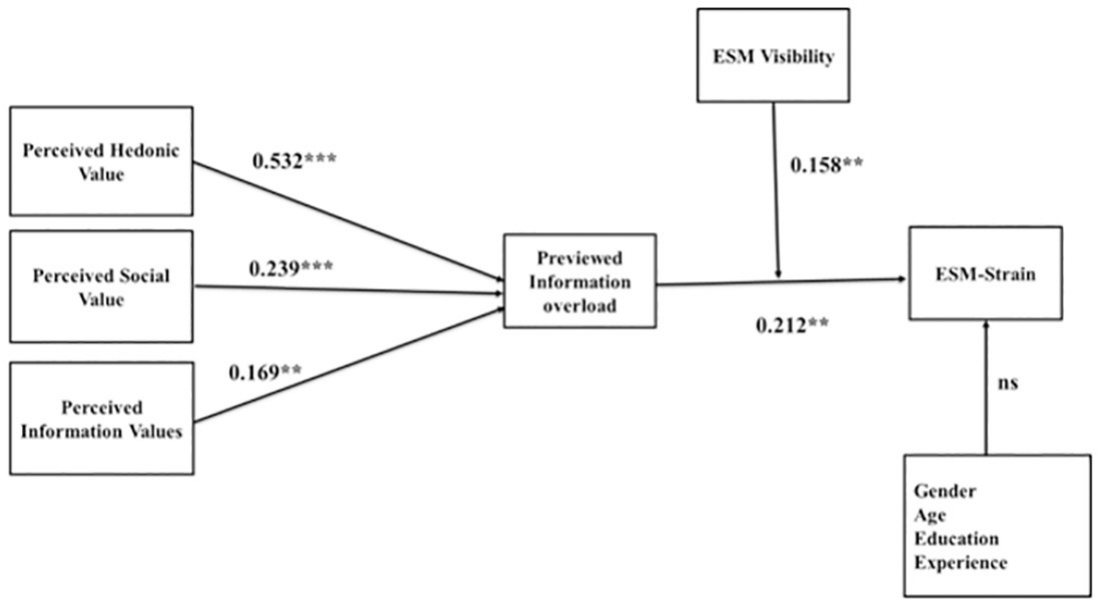
Structural model. Note: *p<0.05, **p<0.01, ***p<0.001.

Table 5 depicts that the outcome of the structural model fit values of all constructs (CFI = 0.903, TLI = 0.885, IFI = 0.930, NFI = 0.870, RMSEA = 0.079, CMIN/DF = 3.520) are all in the recommended range [84, 85]. In addition, Fig 2 indicates the results of path analysis of all the suggested hypotheses. These results indicate that perceived hedonic value (B = 0.532, t = 8.434, p<0.001), perceived social value (B = 0.239, t = 4.275, p<0.001), and perceived information value (*B* = 0.169, *t* = 3.049, *p*<0.01) all have a significantly positive relationship with perceived information overload, thereby supporting H1a, H1b, and H1c. In addition, the results also indicate that perceived information overload has a significant effect on ESM-strain (*B* = 0.212, *t* = 2.592, *p*<0.01), thereby validating H2. Fig 2 also indicated that all the control variables have an insignificant relationship with ESM-related strain.

### 5.3 Moderation analysis

The existing study also analyzed the moderating effect of ESM visibility on the link between perceived information overload and ESM-strain. We proposed in Hypothesis 3 that ESM visibility strengthens the relationship between perceived information overload and ESM train. The findings indicate that the interaction term (perceived information overload × ESM visibility) has a significant relationship with ESM-strain (*B* = 0.113, *t* = 2.011; *p* < 0.05), validating H5a.

**Fig 3:**
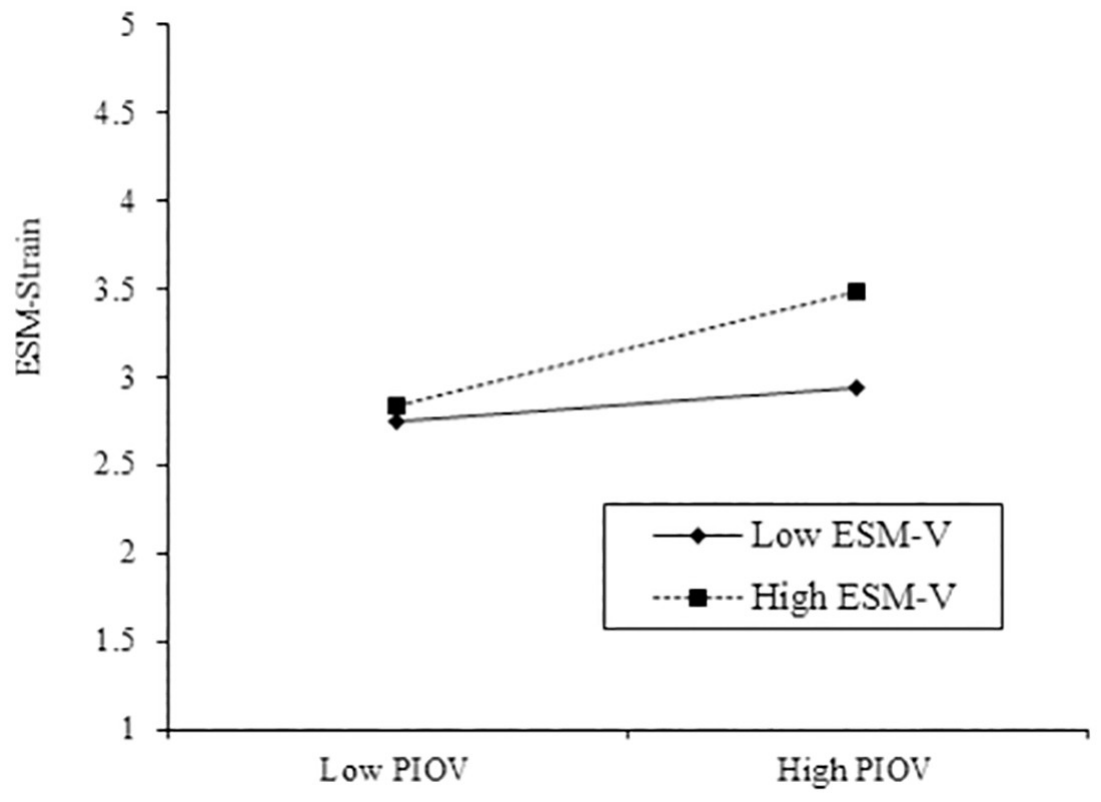
Moderating effect of ESM visibility in the relationship between perceived information overload and ESM-strain. Note: PIOV = Perceived Information Overload, ESM-V = ESM-Visibility.

To fully understand the moderating effect of ESM visibility in our research model, we further used a graphic approach suggested in previous research [86]. According to Fig 3, ESM visibility strengthens the relationship between perceived information overload and ESM strain.

## 6. Discussion, implications, limitations

### 6.1 Discussion

This study investigated ESM-related stain using UGT as a theoretical foundation. It also examined the moderating role of ESM visibility in the link between perceived information overload and ESM-related strain. The empirical analysis validated the suggested hypotheses. Specifically, the results indicated that perceived hedonic, social, and information value have a significant effect on perceived information overload, supporting H1a, H1ab, and H1c, which accords with our assumptions. These findings reflect that, to obtain a high perception of information, social, and hedonic value, employees are likely to share information, ideas, and interactions on the ESM platform with colleagues. Previous studies also reported similar results [12, 24, 87]. For example, Cao and Yu [12] reported that excessive use of ESM has a significant effect on strain. Sun, Liu [8] also observed that perceived hedonic, social, and information value causes excessive usage of ESM by employees. The results also confirmed that perceived information overload has a significant effect on ESM-related strain, thus affirming H2. ESM is an open platform where employees can easily share and exchange work or non-work-related information; this high frequency of information causes anxiety and depression among employees. Previous scholars have also found that information overload has a negative impact on individual work performance [12, 51]. Thus, Chen and Wei [17] also found a curvilinear relationship between information overload and ESM-related strain.

Furthermore, the findings also show that ESM visibility strengthens the relationship between perceived information overload and ESM-related strain—H3 is also supported by current data set. ESM visibility forces individuals to establish a better social presence on ESM, which can necessarily require workers to share and exchange a significant amount of information with other colleagues [65], leading to information overload. ESM visibility also allows employees to view the communication content of others, even if they are not directly involved in that communication [3, 20]. Chen and Wei [17] reported that communication visibility significantly strengthens the relationship between ESM use and information overload.

### 6.2 Theoretical implications

The current study can make numerous theoretical contributions. Firstly, prior studies have been generally based on the positive role of ESM [3, 44, 66]. For example, Pitafi, Kanwal [44] observed that it has a positive effect on individual work performance through task interdependence. Cao, Ali [88] also argued that ESM use enhances team performance. In contrast, the present research investigates the use of ESM and establishes an empirical link between ESM use (hedonic, social, and information values) and ESM-related strain through perceived information overload. These findings further advance our theoretical understanding of the relationship between ESM use and related strain through perceived information overload, clarifying that information overload causes ESM-related strain. A second contribution is that the study’s results also indicate that information overload has a significant effect on ESM-related strain. These results extend those studies that address ESM-related strain in the workplace [36]. We have also highlighted the role of perceived information overload, which exerts a great negative influence on the strain.

Thirdly, the present study investigates the role of ESM visibility as a moderator and found that ESM visibility reinforces the relationship between perceived information overload and ESM-related strain. Previous studies have highlighted the significant role of ESM visibility [3, 65], with Engelbrecht, Gerlach [65] reporting that ESM visibility may have a positive effect on knowledge-sharing because employees can learn from the communication activities of colleagues. Visibility allows individuals to view the historical communication of other employees at any time. Nevertheless, due to nature of ESM technology, ESM visibility also causes information overload.

Finally, this study has some contributions to UGT literature. Although several researchers have used UGT to analyze the formulation mechanism of social media acceptance and continued usage behavior, its impact on the formation process of ESM-related strain has received little consideration. Even if ESM usage in the workplace is not extreme, employee output can suffer as a result of perceived information overload.

### 6.3 Managerial implications

The results of this study have several implications and suggestions for managers. Its findings show that ESM usage (hedonic, social, information value) has a positive effect on perceived information overload since social media is commonly utilized by individuals in corporations for socialization rather than work-related collaboration [17]. Furthermore, the current study indicates that perceived information overload has a significant impact on strain. We suggest that managers acquaint themselves with the features of ESM before applying it within the organization, and guide workers in the logical use of ESM technology for information-sharing. Managers can also apply some policies to control employee’s ESM use. An example is formulating some ESM guidelines that are consistent with corporate culture and specifying when and how an individual can use ESM. Organizations should designate a certain time for workers to use ESM for suitable enjoyment and entertainment, allowing them to better apply themselves to their respective duties.

In addition, this study shows that ESM visibility strengthens the connection between perceived information overload and ESM-related strain. ESM designers can implement some relevant technological features when developing and improving ESM technology. They should specifically strengthen the technological functions relating to ESM visibility or incorporate certain configuration options so that users can easily track and manage their actions and minimize possible excessive usage. For example, designers may include optional features in ESM to limit interaction requests at specific times, thereby reducing the duration and intensity with which workers maintain social queries during work time. ESM developers may also include some screening features that help users to better locate required information on ESM, reducing the amount of time they waste in searching non-related content on ESM during work time.

Furthermore, managers may also provide trainings to their employees to use ESM appropriately. We recommend that individuals can better control and manage their ESM usage by recognizing the causes of excessive use. Accordingly, employees may limit their use of ESM for non-work-related activities during working hours. They should also monitor the extent and duration of their use of ESM at work, as well as using alternative methods of communication such as telephone or face-to-face communication, thus minimizing their dependence on ESM.

### 6.4 Limitations and future directions

Although the current study has numerous implications, there are some limitations that we note here for future researchers. Firstly, the participants of study were Chinese employees and it focused on ESM users. Future scholars may apply the same conceptual model to other countries and compare the results. Nevertheless, China is an ideal country for this study, as ESM is widely used by Chinese employees for work-related communication [8]. In addition, the role of ESM in Chinese culture to satisfy individual social, information, and hedonic desires can vary from Western societies. As a result, future studies may enhance the generalizability of research by incorporating diverse cultural contexts. Another sampling issue is that self-reporting of data by users is used in this study, which is considered subjective [89]. Future scholars can concentrate on various data sources, such as objective data from the technical department on the practical application of ESM.

Thirdly, the existing study is based on a cross-sectional method to demonstrate the impact of visibility allowed by ESM on workers’ excessive usage. A longitudinal method could more deeply reveal the changes in ESM usage behavior over time, which could be more significant. In addition, the current study does not investigate the mediating effect of perceived information overload. Future studies could use another moderator and also examine the mediating role of information overload.

Finally, the present research is an empirical investigation into ESM. While ESM can only be accessed by employees inside the corporation, they also use external social media tools (Twitter, Facebook, Whatapp, WeChat) to not only communicate with workmates but also with friends and relatives [45]. As a result, future studies should also explore how various overload experiences are induced by external social media technology.

## 7. Conclusion

The objective of this study is to investigate the link between the gratification of needs and ESM-related strain through perceived information overload using data collected from Chinese employees. The current study supported all the suggested hypothesis. Specifically, the results indicate that perceived hedonic, social, and information values are significant predictors of perceived information overload. Such overload is also significantly associated with ESM-related strain. The results also indicate that ESM visibility strengthens the significant relationship between perceived information overload and ESM-related strain.

## Supporting information

S1 Appendix Survey questionnaire. (DOCX)
