# Supplementary material for: Investigating the factors of enterprise social media strain: The role of enterprise social media’s visibility as a moderator
Source: PLoS One. 2022 Mar 8;17(3):e0264726. doi: 10.1371/journal.pone.0264726 (PMC8903258; doi:10.1371/journal.pone.0264726)
Supplement: S1 Appendix — (DOCX) [file pone.0264726.s001.docx]

**Investigating the factors of enterprise social media strain: The role Enterprise Social media visibility as a moderator**

**S1 Appendix. Survey Questionnaire.**

| **Constructs and measurement** | **Scale** | **Source** |
| --- | --- | --- |
| Perceived Social Value  1. Sending information to others using ESM can improve my image.  2. Sharing information with others using ESM can improve my relationship.  3. I can know what is going on in my colleague’s lives via ESM. | 1-5 Scale |  |
| **Perceived Information Value**  1. I accumulate numerous knowledge through ESM users shared information.  2. I can acquire a variety of information from colleagues using ESM.  3. I obtain useful information from colleagues using ESM.  4. Overall the last one month, I consulted colleagues using ESM for practical issues and matters. | Likert 1-5 Scale |  |
| **Perceived Hedonic Value**  1. I have fun interacting with ESM.  2. Using ESM provides me a lot of enjoyment.  3. I enjoy using ESM. |  |  |
| **ESM-Strain**  1. I feel drained from activities that require me to use enterprise social media.  2. I feel tired from my enterprise social media activities.  3. Working all day with enterprise social media is a strain for me.  4. I feel burned out from my enterprise social media activities. |  |  |
| **Perceived Information overload**  1. I am often distracted by the excessive amount of information available to me on enterprise social media.  2. I find that I am overwhelmed by the amount of information I have to process on a daily basis on enterprise social media.  3. There is too much information about my coworkers on enterprise social media so I find it a burden to handle.  4. I find that only a small part of the information on enterprise social media is relevant to my needs. |  |  |
| **ESM visibility**  1. Enterprise social media enable me to see other coworkers ‘answers to other coworkers’ question.  2. Enterprise social media enables me to see who has interactions with particular coworkers.  3. Enterprise social media enables me to see the number of others who have linked to the same content. |  |  |
